# A catalytic membrane approach as a way to obtain sweet and unsweet lactose-free milk

**DOI:** 10.1007/s00449-024-03018-z

**Published:** 2024-04-22

**Authors:** Katarzyna Czyżewska, Anna Trusek

**Affiliations:** Faculty of Chemistry, Group of Micro, Nano, and Bioprocess Engineering, Wroclaw University of Science and Technology, Norwida 4/6, 50-373 Wrocław, Poland

**Keywords:** NOLA Fit 5500, Glucose oxidase, Catalase, Co-immobilization

## Abstract

The growing need in the current market for innovative solutions to obtain lactose-free (L-F) milk is caused by the annual increase in the prevalence of lactose intolerance inside as well as the newborn, children, and adults. Various configurations of enzymes can yield two distinct L-F products: sweet (*β*-galactosidase) and unsweet (*β*-galactosidase and glucose oxidase) L-F milk. In addition, the reduction of sweetness through glucose decomposition should be performed in a one-pot mode with catalase to eliminate product inhibition caused by H_2_O_2_. Both L-F products enjoy popularity among a rapidly expanding group of consumers. Although enzyme immobilization techniques are well known in industrial processes, new carriers and economic strategies are still being searched. Polymeric carriers, due to the variety of functional groups and non-toxicity, are attractive propositions for individual and co-immobilization of food enzymes. In the presented work, two strategies (with free and immobilized enzymes; *β*-galactosidase NOLA, glucose oxidase from *Aspergillus niger*, and catalase from *Serratia sp.*) for obtaining sweet and unsweet L-F milk under low-temperature conditions were proposed. For free enzymes, achieving the critical assumption, lactose hydrolysis and glucose decomposition occurred after 1 and 4.3 h, respectively. The tested catalytic membranes were created on regenerated cellulose and polyamide. In both cases, the time required for lactose and glucose bioconversion was extended compared to free enzymes. However, these preparations could be reused for up to five (*β*-galactosidase) and ten cycles (glucose oxidase with catalase).

**Graphical abstract:**

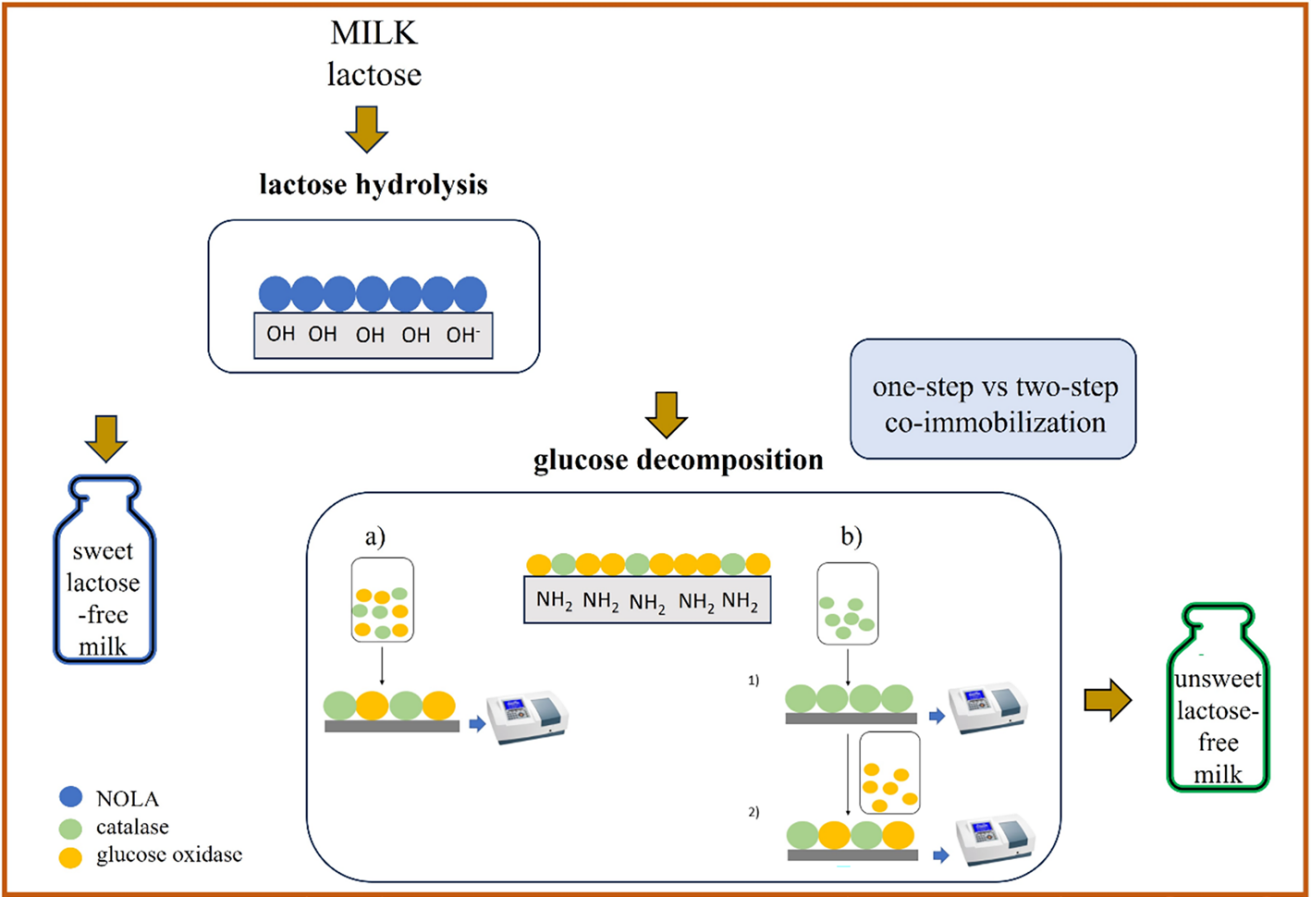

**Supplementary Information:**

The online version contains supplementary material available at 10.1007/s00449-024-03018-z.

## Introduction

Lactose intolerance is a prevalent ailment affecting a significant portion of the global population. According to epidemiological data, up to 70% of the world’s population may be affected by this dysfunction [1]. Lactose intolerance is manifested by inadequate enzyme *β*-galactosidase (*β*-gal) required for lactose hydrolysis in the small intestine. Consequently, consumption of lactose-containing foods (both dairy and non-dairy products) by individuals with *β*-galactosidase deficiency results in gastrointestinal discomfort [2, 3]. One of the easiest ways to deal with lactose intolerance is its elimination from the daily diet by replacing traditional dairy with lactose-free (L-F) products. Nowadays, L-F-labeled dairy products are perceived as functional foods, substitutes for whole milk, and a low-cost dietary source of calcium with a wide range of availability [4, 5].

In recent years, there has been growing interest in finding technologies that provide nutritional value and functional, sensory, and quality properties of L-F dairy products [4]. Among the existing methods for removing lactose from food are membrane separation, fermentation, and enzymatic hydrolysis. The last one has gained high popularity in industrial technologies [4, 6]. It is the result of high specificity and relatively low cost of bioconversion (especially in the case of immobilized preparations) [7]. In the enzymatic approach, two strategies are proposed: batch, in which enzyme is added before pasteurization, and aseptic, in which the sterile enzyme is added to UHT milk before packaging [6].

**Fig. 1:**
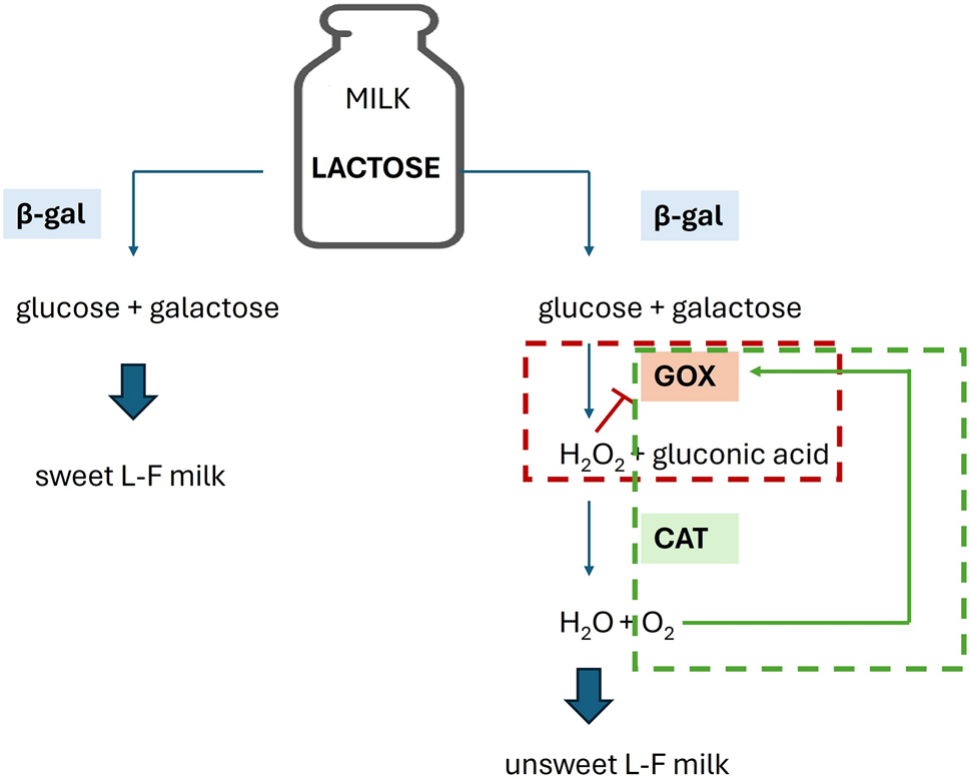
Enzymatic pathways to obtain sweet and unsweet L-F milk. The red lines represent the step with product inhibition caused by H_2_O_2_, and the green lines show benefits related to the presence of catalase in the reaction mixture (decomposition of H_2_O_2_ and generation of O_2_, which acts as a substrate for GOX) (colour figure online)

Enzymatic lactose hydrolysis allows the production of two kinds of L-F milk: sweet and unsweet, Fig. 1. To get the first, the one-enzyme pathway with *β*-galactosidase is necessary. The higher sweetness of L-F milk is caused by the products of lactose hydrolysis, glucose, and galactose, both of which have a higher sweetness index than lactose. Therefore, L-F milk is gaining popularity in producing low-calorie dairy desserts without supplementary sweeteners [8]. Receiving unsweet L-F milk is more complicated and involves three enzymatic pathways [9]. Because the noticeable sweetness of L-F milk is mainly the result of glucose [10], its decomposition by glucose oxidase (GOX) is desirable [11]. Although catalase (CAT) does not directly participate in the decomposition of glucose, its presence, mainly in the one-pot mode, is necessary to maintain the high enzymatic activity of GOX inhibited by hydrogen peroxide. Unsweet L-F milk is dedicated to an increasingly more extensive group of consumers who negatively perceive the sweetness of L-F milk [12]. Furthermore, unsweet L-F milk, due to its similar taste to traditional milk, can be applied in savory dishes and compete with non-dairy milk alternatives [13].

Industrial biocatalysis is closely related to enzyme immobilization, helping to overcome the limitation of free enzymes [14, 15]. Immobilized preparations created through chemical, physical or physico-chemical methods bring the benefits of improved enzyme stability, easy separation from the medium, reusability, and use in continuous processes [15, 16]. The enzyme properties and future application determine the choice of an appropriate carrier. Beyond the commonly known properties of classic carriers utilized for enzyme immobilization, such as non-toxicity, biocompatibility, and insolubility, food processes also require high chemical stability and resistance to microorganisms [14].

Among the wide range of carriers dedicated to industrial biocatalysis, polymeric membranes are highly valued [17–19]. They can serve as a separating layer in enzymatic membrane reactors, enabling the retention of free enzymes within the reactor space or functioning as carriers for immobilized enzymes (catalytic membranes) [20].

The standout feature of polymeric membranes lies in their high surface area, which facilitates enzyme immobilization either on the surface or within the membrane’s pores. In addition, their robust mechanical strength and modifiable surface chemistry make them conducive to enhancements in immobilization efficiency [20]. Enzymes can be immobilized onto polymeric membranes through physical adsorption or covalent binding. Establishing chemical bonds between the polymeric membrane and enzyme molecules facilitates the development of highly efficient catalytic membranes, minimizing the risk of enzyme leakage. When evaluating catalytic membranes, priority should be given to characterizing the enzyme surface concentration, while their separating properties may be considered secondary [21, 22].

Nowadays, catalytic membranes are not limited to one-enzyme preparations. The variety of functional groups on the membrane surface facilitates the creation of co-immobilized preparations [23, 24]. Their application is particularly recommended for biocatalytic processes with inhibition by-product [25] Bioconversion based on co-immobilized enzymes limits the accumulation of side products, decreases the number of steps of the process (one-pot mode), and reduces the idle time [26].

This study presents two enzymatic pathways to obtain sweet (one-enzyme approach: *β*-gal) and unsweet (three-enzyme approach: *β*-gal, GOX, CAT) L-F milk under low-temperature conditions. Furthermore, lactose hydrolysis and glucose decomposition were carried out with catalytic membranes including one or two immobilized enzymes to enhance the economic profitability of the process, and in response to the demands of the food industry in different lactose-free products. The method proposed in this study tackles the challenge of multi-enzymatic immobilization while mitigating the risk of enzyme leakage, commonly observed in processes involving hydrogels, by employing stable covalent bonding.

## Materials and methods

### Chemicals

The following enzymes were used in the research: *β*-galactosidase NOLA^TM^Fit5500 (NOLA) (Chr. HANSEN), glucose oxidase from *Aspergillus niger* (Merck), and catalase obtained from recombinant microorganisms of the *Serratia* sp. genus (Swissaustral). HEPES buffer, glutaraldehyde (GA), divinyl sulfone (DVS), Lowry’s reagent, regenerated cellulose (CAS 10410214), and polyamide (GNWP02500) membranes were procured from Merck. The manufacturer of the analytical test for determining glucose concentration was Biomaxima. Other reagents were obtained from Avantor Performance Materials Poland S.A. In experiments carried out in real medium, the skimmed raw cow’s milk from the ecological farm Kozia Laka (Lomnica, Poland) was used.

### Biocatalysis with free enzymes to obtain sweet and unsweet L-F milk

The studies were carried out in skimmed raw cow’s milk. To obtain sweet L-F milk, lactose was hydrolyzed by free NOLA at a concentration of 1.5 g/L in a thermostated glass reactor at the working volume of 5 mL (12 °C) and at a mixing speed of 240 rpm. The progress of the reaction was determined by measuring glucose concentration using a Biomaxima analytical test, as previously described by the authors [27]. Briefly, 10 μL of the sample was added to 1 mL of the glucose reagent. The mixture was then incubated for 5 min at 37 °C. Subsequently, the absorbance was measured at 500 nm. The first step to obtain unsweet L-F milk involved lactose hydrolysis by free NOLA. Then glucose decomposition realized in one-pot mode with GOX and CAT was performed. The initial glucose concentration in the reaction mixture was 27.5 g/L. The following enzyme concentrations were used in this case: 2.7 and 0.43 g/L, respectively, for GOX and CAT. Glucose decomposition was carried out at 12 °C. The reaction mixture was aerated with compressed air at an airflow of 4.5 L/min. The solution of 1 M NaOH was used to adjust the pH of the milk to a value of 6.6. The addition of NaOH was carefully controlled and did not exceed 1% of the total milk volume. Emerging foam was broken down mechanically using a self-built foam breaker. The progress of the reaction was monitored based on changes in glucose concentration over time using the DNS (3,5-dinitrosalicylic acid) test, according to the procedure described previously [9]. In this case, 0.5 mL of sample was added to 1.5 mL of DNS reagent. The mixture was incubated for 5 min at 100 °C, followed by cooling. After 25 min, 8 mL of distilled water was introduced. The absorbance of the samples was measured at 550 nm. All measurements were performed in triplicate. The analytical error never exceeded 5%. The graph shows the average values.

### Catalytic membranes: individual immobilization of β-galactosidase, glucose oxidase, and catalase

Enzyme immobilization (NOLA, GOX, and CAT) was performed on two types of membranes, regenerated cellulose (RC, pore diameter 0.45 μm) and polyamide (PA, pore diameter 0.2 μm), both with a surface area of 4.9 cm^2^. The membrane activation and enzyme immobilization procedure were carried out in an Amicon-stirred cell of volume 10 mL at 8 °C. Both actions were taken in a closed circuit, without circulation of the reaction mixture. For the membrane RC, the method previously described by the authors was employed [28]. Briefly, RC membranes were activated with a 10% DVS solution in 1 M Na_2_CO_3_, with a volume of 6 mL, for 2.5 h at 8 °C. After washing with 0.1 M PBS buffer, the enzyme solution prepared in 0.5 M NaHCO_3_/Na_2_CO_3_, pH 9.0, with a volume of 4.5 mL, was introduced into the Amicon cell for 24 h at 8 °C, with agitation at 300 rpm. The unbound enzyme molecules were removed by rinsing the membrane multiple times with PBS buffer. Several modifications have been made to the PA membrane. Before the activation step, the PA membrane was washed with 0.1 M PBS buffer pH 7.0. Then it was activated with a 2.5% GA solution prepared in 0.1 M PBS buffer in a volume of 6 mL for 1 h, 8 °C. After that, the membrane was washed with 0.1 M PBS, pH 7.0, to remove excess of the coupling agent. The enzyme solution prepared in 0.1 M PBS buffer with a volume of 4.5 mL was then added to the membrane surface. Enzyme immobilization lasted 24 h at 8 °C. The unbound enzyme molecules were removed by rinsing the membrane multiple times with PBS buffer. The immobilization yield was determined as previously, based on the protein balance by measuring protein concentration before and after immobilization using the Lowry method [29]. All measurements were performed in triplicate and the average values are presented. The supplied masses of enzymes during immobilization were as follows: NOLA (RC membrane) 2.08–50.86 mg, GOX (RC membrane) 0.97–16.54 mg, CAT (RC membrane) 0.95–2.61 mg, GOX (PA membrane) 0.51–30.45 mg, CAT (PA membrane) 0.44–2.44 mg.

### Catalytic membranes with co-immobilized glucose oxidase and catalase

One-step co-immobilization of GOX and CAT was performed on the PA membrane. The immobilization procedure was identical (with one change) to that described for individual immobilized enzymes. During immobilization, the mixture of both enzymes containing 0.90–4.00 g/L of GOX and 0.34–0.68 g/L of CAT was supplied to the surface of the activated membrane. The mass of enzymes was calculated as 1.46–5.80 mg. After immobilization, enzyme binding efficiency was determined based on Lowry’s method and protein balance. On the one-step approach, immobilization yield was referred to as the total mass of GOX and CAT attached to the membrane.

Two-step co-immobilization on the PA membrane was performed according to the procedure described for individually immobilized enzymes. First, the GOX solution was added to the membrane surface. When immobilization was complete, Lowry’s method determined binding efficiency based on the protein balance. The CAT solution was applied to the activated membrane with bounded GOX in the second step. The efficiency of CAT binding was determined according to Lowry’s method. All measurements were performed in triplicate and the average values are presented. The final immobilization effectiveness referred to the total bound mass of GOX and CAT, in which the mass ratio of both enzymes could be determined. The concentration of enzymes during immobilization was in the range of 0.56–1.12 and 0.28–0.56 g/L for GOX and CAT, respectively. The value of the supplied mass of enzymes was 1.26–2.98 and 1.05–1.30 mg for GOX and CAT.

### Lactose hydrolysis and glucose decomposition catalyzed by catalytic membranes

Lactose hydrolysis and glucose decomposition were carried out in thermostated glass reactors (12 °C, mixing speed 240 rpm), within which catalytic membranes were mounted at the bottom of the reactor. Lactose solution at a concentration of 55.0 g/L and glucose at a concentration of 27.5 g/L was prepared in 0.1 M HEPES buffer, pH 6.6. The progress of bioconversions was monitored based on changes in glucose concentration over time, determined by the Biomaxima analytical test (lactose hydrolysis) or by DNS reagent (glucose decomposition). All measurements were performed in triplicate and the average values are presented.

### Reusing of catalytic membranes

The possibility of reusing catalytic membranes was expressed as cycles that ensure more than 90% lactose bioconversion yield (for membrane with NOLA) and glucose bioconversion (for membrane with GOX and CAT). The reaction mixture consisted of a lactose solution at a concentration of 55.0 g/L prepared in 0.1 M HEPES buffer, pH 6.6 or a glucose solution at a concentration of 27.5 g/L prepared in 0.1 M HEPES buffer, pH 6.6. The bioconversion of lactose and glucose was carried out in a thermostated glass reactor at 12 °C. The progress of biocatalysis was monitored based on changes in glucose concentration over time, as described above. All measurements were performed in triplicate and the average values are presented.

## Results

### Utilizing free enzymes to obtain sweet and unsweet L-F milk

To obtain sweet L-F milk, the one-enzyme approach with free NOLA was proposed. The second product, unsweet L-F milk, was achieved in the one-pot mode, with GOX and CAT. Although CAT does not directly participate in glucose decomposition, its presence was necessary to maintain the high enzymatic activity of GOX, through the decomposition of H_2_O_2_, which acts as inhibitor toward GOX [9]. The proposed strategy referred to previous results, in which authors discussed the importance of product inhibition during NOLA-induced lactose hydrolysis [27] and indicated the critical process parameters (pH of medium and oxygen concentration) that accompany the enzymatic decomposition of glucose by a two-enzyme cascade with GOX and CAT [9].

**Fig. 2:**
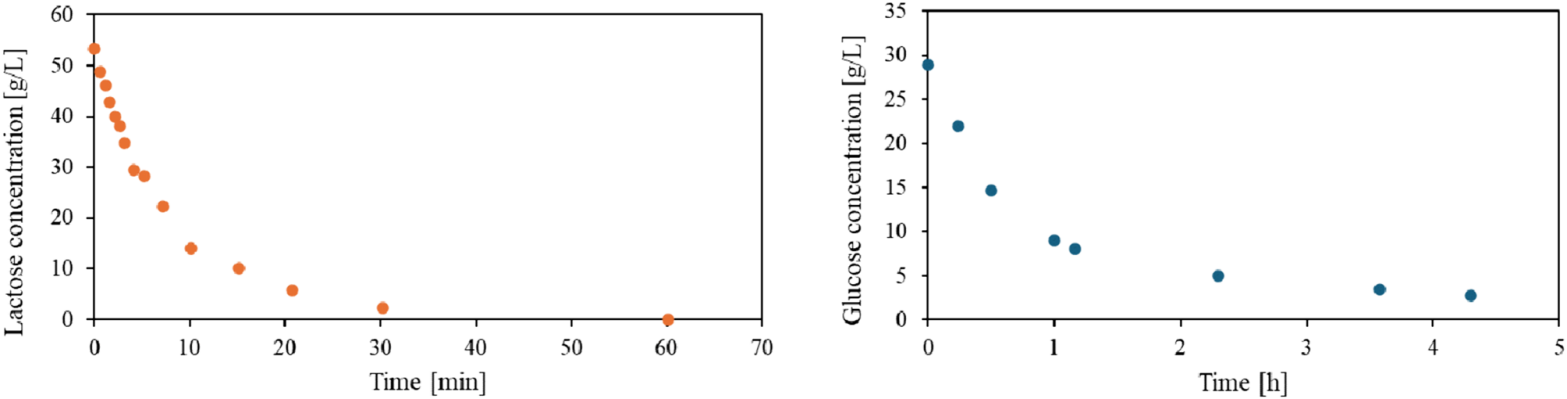
Enzymatic approach employing free enzymes to obtain sweet L-F milk based on NOLA-induced lactose hydrolysis (*left*) and unsweet L-F milk based on glucose decomposition by GOX and in the presence of CAT (*right*). Lactose hydrolysis was tracked by observing alterations in glucose concentration. Skimmed raw cow’s milk, initial lactose concentration in milk 55.0 g/L, glucose in L-F milk 26.72 g/L, NOLA 1.5 g/L, GOX 2.7 g/L, CAT 0.43 g/L 12 °C

Enzymatic hydrolysis of lactose catalyzed by NOLA lasted 60 min at 12 °C and allowed to obtain milk with a lactose concentration of less than 0.01% (m/v), Fig. 2. The final product, sweet L-F milk, was enriched with glucose at a concentration of 26.72 g/L. The distinct sweetness of L-F milk was confirmed organoleptic. However, its aroma and color resembled those of conventional cow’s milk [27].

The previously obtained sweet L-F milk was used to receive unsweet L-F milk. The step involving sweetness reduction by glucose decomposition was performed in one-pot mode with GOX and CAT and with continuous aeration of the milk with compressed air, at 12 °C. Due to its rapid removal by a mechanical foam breaker, the foam generated during aeration did not hinder biocatalysis. To adjust the pH of L-F milk close to 6.6, the solution of 1 M NaOH was utilized. Glucose decomposition to an acceptable level (conversion ≥ 90%) lasted 4.3 h, Fig. 2. The parameters proposed to obtain two types of L-F milk, sweet and unsweet, are detailed in Table 1.

**Table 1:** Process parameters proposed to obtain sweet and unsweet L-F milk by free NOLA, GOX and CAT, at 12 °C

	Sweet L-F milk	Unsweet L-F milk
	Raw milk	Sweet L-F milk
Initial lactose conc. [g/L]	55.0	0.08
Initial glucose conc. [g/L]	–	26.72
NOLA [g/L]	1.5	–
GOX [g/L]	–	0.3
CAT [g/L]	–	1
T [°C]	12	12
Conversion degree ≥ 90% [h]	1	4.3
pH regulation	–	NaOH
Aeration	–	compressed air

### Catalytic membranes design

The results presented for free enzymes were the basis for the creation of catalytic membranes. Due to the generation of two value-added products, sweet and unsweet L-F milk, two distinct preparations were developed: with covalently immobilized NOLA and covalently co-immobilized GOX and CAT. Regardless of the immobilization strategy, one or two enzymes, the formation of catalytic membranes was preceded by individual immobilization of the enzymes tested. Experiments with catalytic membranes were conducted in a buffered solution of lactose and glucose, referring to the previously results, in which the bioconversion of lactose and glucose was compared with buffered solutions and real medium [9].

### Catalytic membranes with β-galactosidase NOLA

NOLA immobilization was performed on a DVS-activated RC membrane. The efficiency of NOLA binding through the hydroxyl groups of regenerated cellulose was analyzed in a wide range of enzyme concentrations: 0.46–24.67 g/L. Consequently, the optimal value for the supplied mass of NOLA was chosen. It was close to 40 mg, Table 2.

**Table 2:** Efficiency of the creation of catalytic membranes with NOLA

	RC membrane				
Supplied mass of enzyme [mg]	2.08	12.61	39.92	41.75	50.86
Surface concentration [g/m^2^]	0.3	1.73	6.49	4.61	5.08
Immobilization efficiency [%]	8.65	6.71	7.97	5.42	4.90

**Fig. 3:**
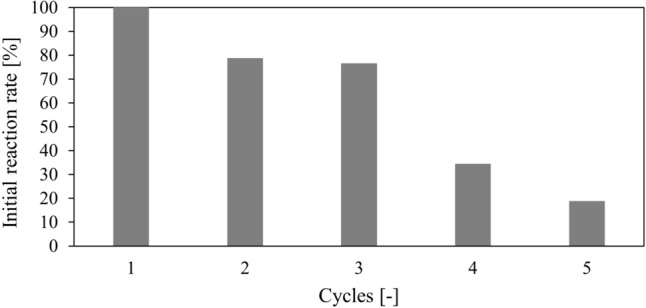
Reuse of RC catalytic membrane with NOLA. The reference value of the initial reaction rate was 0.009 g/(L min). Lactose 55.0 g/L in HEPES, surface concentration of NOLA 6.49 g/m^2^, *V* = 5 mL, *A* = 4.9 cm^2^, time of 1 cycle 48 h

One of the most significant advantages of immobilized enzyme preparations is their potential for reuse in subsequent reaction cycles. Catalytic membranes with immobilized NOLA could have been used for five consecutive reaction cycles, retaining 20% of their initial activity, Fig. 3. Each reaction in the cycle lasted 48 h. The reaction time in the final cycle was extended to 90 h. Considering the efficiency of NOLA immobilization on the RC membrane (Table 2), we explored the potential for reusing the enzyme solution that was applied to the membrane surface to create subsequent catalytic membranes. These results are presented in the Supplementary Information, Fig. S1.

NOLA immobilized on a GA-activated PA membrane was inactive. The same effect was achieved for various concentrations of GA, Supplementary Information, Tables S1 and S2.

### Catalytic membranes with co-immobilized glucose oxidase and catalase

The individual immobilization of both enzymes preceded the creation of a co-immobilized catalytic membrane with GOX and CAT. Utilization of a DVS-activated RC membrane caused an irregular correlation between the supplied mass of GOX and CAT and obtained surface concentration of enzymes, negatively affecting immobilization efficiency, which is presented in Supplementary Information, Figs. S2 and S3.

Therefore, subsequent experiments were carried out on a GA-activated PA membrane. In this case, the different degrees of GOX and CAT affinity to the amino groups of polyamide were observed. The higher binding efficiency exhibited catalase, Table 3.

**Table 3:** Efficiency of the creation of catalytic membranes with GOX and CAT

	PA membrane	
	GOX	CAT
Supplied mass of enzyme [mg]	0.51–30.45	0.44–2.24
Surface concentration [g/m^2^]	0.4–2.24	0.25–1.73
Immobilization efficiency [%]	7.84–12.89	27.27–37.95

The differences in the degree of GOX and CAT affinity for the functional groups of the polyamide membrane led to the formulation of two distinct strategies for creating co-immobilized membranes: the one- and two-step co-immobilization.

In both variants, the ratios in enzyme concentration were a crucial parameter. In the one-step approach, immobilization efficiency was determined by the total mass of the bound enzymes, while in the two-step approach, it was specified by the mass of each enzyme. Regardless of the chosen strategy, an increase in the total mass of bound enzymes was observed only in variants characterized by lower concentrations of GOX. These observations confirm glucose oxidase’s limited binding capacity with polyamide’s amino groups. The effectiveness of producing co-immobilized catalytic membranes with GOX and CAT has been summarized in Table 4.

**Table 4:** Efficiency of the creation of co-immobilized catalytic membranes with GOX and CAT based on one- and two-step co-immobilization

	Polyamide		
	Supplied mass of GOX [mg]	Supplied mass of CAT [mg]	Surface concentration [g/m^2^]
One-step co-imm	1.46–5.8		0.67–1.69
Imm. efficiency [%]	14.31–22.6		
Two-step co-imm	1.26–2.98	1.05–1.3	0.78–1.14
Imm. efficiency [%]	4.03–6.35	27.62–31.54	

**Fig. 4:**
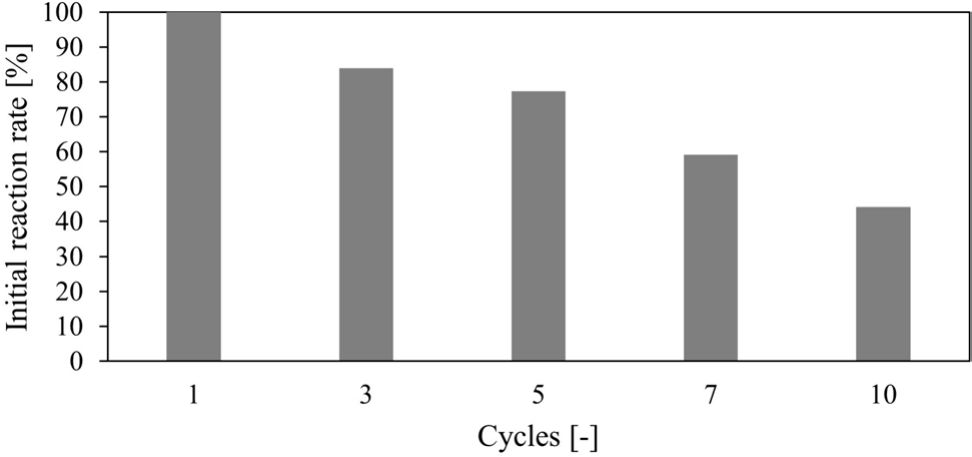
Reuse of catalytic membranes with co-immobilized GOX and CAT prepared based on one-step co-immobilization. The reference value of the initial reaction rate was 1.014 g/(L min.). Glucose 27.5 g/L in HEPES, total surface concentration 1.69 g/m^2^, *V* = 3 mL, *A* = 4.9 cm^2^, CaCO_3_, saturation with compressed air

The presented results indicate no significant differences between both strategies, one- and two-step co-immobilization. The most significant advantage that favors the one-step approach is the simplicity of execution. According to that, the membranes prepared in one-step mode were used to evaluate their potential for reuse, Fig. 4. These preparations could be utilized for ten consecutive cycles, retaining 44% of their initial activity. Each reaction in the cycle lasted 24 h. The reaction time in the final cycle was extended to 45 h. Table 5 compares lactose and glucose decomposition catalyzed by the free and immobilized enzymes.

**Table 5:** Lactose and glucose decomposition catalyzed by the free and immobilized enzymes

	Free enzymes		Catalytic membranes	
Substrate	Lactose	Glucose	Lactose	Glucose
Conversion time (*α *> 90%) [hours]	1	4.3	48	24
Enzyme mass in reaction [mg]	7.5	9	2.26	0.83

## Discussion

### Utilizing free enzymes to obtain sweet and unsweet L-F milk

Enzymatic lactose hydrolysis influenced by the number of enzymes employed in bioconversion enables one to obtain two dairy products with significantly reduced lactose content: sweet and unsweet L-F milk. Both respond to the preferences of consumers, who, on the one hand, want to consume dairy desserts without external sweeteners and, on the other hand, appreciate the authentic taste of traditional milk [12].

Independently of the final product, sweet or unsweet L-F milk, a critical stage enabling the design of high-efficiency bioconversion of lactose and glucose at low temperatures is an appropriate selection of enzymes. The utilized enzymes: *β*-galactosidase NOLA from *Bifidobacterium bifidum*, glucose oxidase from *A. niger*, and catalase from *Serratia sp*., exhibited the preferred properties of dairy enzymes, such as safe for health, higher solubility, high product yield, high stability in a wide range of pH, low temperature and under specific operating conditions, e.g., aeration [30]. Furthermore, their activity at 12 °C and source of isolation—microorganisms Generally Recognized As Safe—correspond to the industrial requirements of food processes and ensure the preservation of the nutritional value of the processed raw materials [14]. These observations were confirmed by literature studies on dairy processes and low-temperature biocatalysis [9, 27, 31–33].

Industrial biocatalysis attempts to find a balance between the conversion time and the dose of the added enzymes. Similar assumptions were incorporated into the presented results. The obtained time (1 h) required to reduce lactose concentration in milk to the level indicated in legal regulations (<0.01% m/v) [6] seems to be satisfactory, especially when contrasted then with the results of other authors who utilized *β*-galactosidase NOLA to lactose hydrolysis at much higher temperatures (35–50 °C) [34, 35]. Furthermore, the recommended concentration of NOLA (1.5 g/L) allowed the performance of lactose hydrolysis in milk without product inhibition caused by both glucose and galactose [27]. The proposed strategy to obtain unsweet L-F milk involved the one-pot mode with GOX and CAT. According to previously presented studies, CAT was necessary to decompose H_2_O_2_ and thus eliminate product inhibition. The amount of generated H_2_O_2_ was enough to inhibit GOX but insufficient to supply the adequate amount of oxygen that ensures the high activity of GOX. Therefore, the external aeration was applied [9]. The presence of CAT allowed the reduction of the time of glucose decomposition to 4.3 h at 12 °C. The benefits related to the cooperation of CAT and GOX, primarily when they work in one-pot mode, were also confirmed by other authors [36, 37]. According to the presented strategy to obtain sweet and unsweet L-F milk, the enzymatic hydrolysis of lactose and glucose decomposition could occur even during milk storage, with a temperature close to 12 °C [38].

The proposed unsweet L-F milk has been enriched with sodium gluconate, a by-product formed in the presence of gluconate acid and NaOH that was used to regulate the pH of the milk. The presence of sodium gluconate in milk should not raise concerns for two reasons: NaOH is a widely accepted food additive approved by the FAO (Food and Agriculture Organization of the United Nations) [39], and sodium gluconate offers the benefits of preventing the formation of calcium lactate crystals [40].

### Catalytic membranes with β-galactosidase NOLA

Hydrophilic polymers, like regenerated cellulose or polyamide, allow the creation of stable enzyme-membrane binding and, thus, the formation of highly efficient catalytic membranes. Most are formed based on covalent bonds engaged in reactive groups located on the membrane surface and active functional groups of enzymes [41]. The results validate the promising potential for creating catalytic membranes with *β*-galactosidase NOLA immobilized on regenerated cellulose. Their activity under low-temperature conditions (12 °C) meets a crucial requirement for dairy biocatalysis.

The rate of lactose hydrolysis depends on the surface area of the RC membrane, which relates to the number of functional groups (OH), which in turn translates into the number of immobilized enzyme molecules. In laboratory studies, the membrane packing was low at only 1.63 cm^2^/cm^3^, while in industrial modules with micro, ultrafiltration membranes, this value reaches several hundred times more [42, 43]. Increasing the surface area of the membranes will have a positive (directly proportional) effect on reducing the catalysis time, which may be only a few tens of minutes.

The obtained results suggested that the immobilization of NOLA on the polyamide membrane was not advantageous. Findings from other studies have shown that the efficiency of immobilizing *β*-galactosidase is strongly influenced by the enzyme’s isolation source, particularly in covalent binding with glutaraldehyde [44]. In addition, the immobilization of *β*-galactosidase presented in this study was conducted randomly, which, as Bhattacharyyaa and coworkers have pointed out, is likely less effective than site-directed immobilization [45].

The favorable results in creating catalytic membranes with *β*-galactosidase have also been documented for membranes composed of cellulose acetate and polymethylmethacrylate [46] or polyvinylidene fluoride [47]. It is worth highlighting the simplicity of the proposed protocol, recommended for creating a catalytic membrane on RC, in which a renewable, economically attractive, and easily accessible material served as a carrier. Moreover, no specific documentation characterizing catalytic membranes with covalently immobilized NOLA exist in the available literature. Instead, covalent immobilization on chitosan beads is proposed [48].

### Catalytic membranes with co-immobilized glucose oxidase and catalase

Enzyme co-immobilization intensifies industrial biocatalysis due to a combination of two or more-unit operations. Polymeric membranes, due to the abundance of surface-located functional groups and flat shape, promote the formation of stable covalent bonds and effective co-immobilized catalytic membranes [23]. Developing a co-immobilized preparation must consider the unique affinities of each enzyme for establishing long-lasting connections with the carrier. Therefore, the individual immobilization of enzymes offers valuable insights. The obtained results referred to the individual immobilization of GOX and CAT on the PA membrane, revealing differences in the affinity of enzymes to amino groups of polyamide. The lower binding affinity of GOX to amino groups contrasted with the findings in the literature. Some reports suggest the successful immobilization of GOX on nylon membranes using amino groups [49, 50], but others offer a poorer immobilization efficiency by covalent bonding on the PDA coating membrane [51]. The satisfactory results of the covalent immobilization of catalase on polyamide have also been confirmed by literature reports [52, 53].

The proposed covalent immobilization of GOX and CAT onto RC membrane via hydroxyl groups exhibited lower efficiency compared to PA membrane. It is worth noting that the GOX bond involving glutaraldehyde, as highlighted in literature reports, is superior. This bond suggests the conversion of originally occurring hydroxyl groups of PMMA microspheres to aldehyde groups, a crucial step for efficient GOX immobilization [54]. Other references indicated the function of RC membrane solely as a separating layer for free GOX and CAT, allowing enzyme retention in the reactor space [55, 56].

It is essential to recognize the versatility of the two distinct unit processes—lactose hydrolysis for producing sweet L-F milk and glucose decomposition for obtaining L-F milk with a traditional taste. This versatility is a testament to the potential of using two different carriers for immobilization (RC membrane for NOLA and PA membrane for GOX and CAT), which should not be seen as an obstacle to future industrial processes.

The presented results indicate the high stability of catalytic membranes with co-immobilized enzymes onto polyamide. These results are notably intriguing since a polyamide membrane has not been widely discussed in the literature as a preferred carrier for the co-immobilization of GOX and CAT. Instead, the proposition of an ultrafiltration membrane composed of an acrylonitrile copolymer [57] or an ultrafiltration membrane made of polypropylene with a skin layer of RC can be found [58]. Polyamide materials, available in membranes, gels, and non-wovens, are widely acknowledged as popular carriers for enzyme immobilization. These materials gained popularity due to their porous structure, chemical and thermal resistance, resistance to biodegradation, and mechanical properties that enable them to withstand increased pressure in membrane flow bioreactors [59]. Some polyamide carriers require partial hydrolysis of the membrane surface to generate more reactive amine groups necessary for enzyme immobilization [60]. Examples of immobilized preparations created on polyamide (PA) and tailored for industrial applications include laccase bonded on functionalized polyamide 6,6 with an immobilization yield of 2% [60], *β*-xylosidase bonded on a microfiltration polyamide membrane with an immobilization yield of 20% [61], and laccase bonded on polyamide fabric hydrolyzed by bromelain with an immobilization yield of 68% [62].

The literature review focusing on carriers dedicated to GOX and CAT co-immobilization highlights the potential of the one-step strategy of co-immobilization onto polyamide membranes, which was presented in this work. Furthermore, the results suggest achieving a higher value of the bonded mass of enzymes in co-immobilized preparations, exceeding 0.83 mg to accomplish a bioconversion time close to free enzymes. This goal, whether for one-enzyme or two-enzyme immobilization, can be achieved, e.g., by increasing the surface area of the carrier.

The proposed strategy of immobilizing NOLA, GOX, and CAT via covalent binding on microfiltration membranes (RC and PA) was less effective than encapsulated preparations previously described by the authors [9, 27]. The lower activity of the catalytic membranes may be attributed to enzyme denaturation upon immobilization, changes in active-site conformation, and crowding of enzyme molecules on the carrier [45]. Despite the higher enzymatic activity of encapsulated preparations, their utilization in continuous processes, such as in packed bead reactors, may pose difficulties in maintaining a constant porosity of the deposit (alginate beads). Furthermore, the comparison of enzymes reused in subsequent cycles for encapsulated preparations and catalytic membranes with NOLA, GOX, and CAT [9, 27] indicated a decrease in enzymatic activity in each successive cycle, regardless of the form of the immobilized preparation, suggesting enzymes’ instability under process conditions.

## Conclusion

The market potential for L-F products is still growing. Thanks to the enzymatic approach and immobilization technique, lactose hydrolysis can be recognized as an efficient and economical method for obtaining two types of L-F milk (sweet and unsweet) at low temperatures, similar to those used in storing and transporting raw milk. Catalytic membranes featuring individually immobilized enzymes and co-immobilized preparations are a prime illustration of multidisciplinary technology that aligns with the increasing demand for highly efficient biocatalysis in the dairy industry. However, creating catalytic membranes without the time and cost-consuming modification of membrane surface chemistry remains challenging. Fundamental limitations are associated with the low affinity of enzyme molecules to functional groups of the membrane and the risk of excessive stiffening of the spatial structure of the enzyme. Nevertheless, the stable connection of the enzyme with the carrier via covalent interactions enabled the catalytic membranes to be reused multiple times. In addition, the high mechanical stability of catalytic membranes allows their application in continuous industrial processes, offering significant opportunities for optimization at various stages. Nonetheless, the development of novel, cost-effective immobilization strategies that ensure high catalyst loading, elevated activity, and long-term stability of catalytic membranes remains a persistent challenge highlighting the need for ongoing research and innovation in this field.

### Supplementary Information

Below is the link to the electronic supplementary material.

Supplementary file1 (DOCX 122 KB)

## Data Availability

All data generated or analyzed during this study are included in this published article (and its supplementary information files).
