# Supplementary material for: A catalytic membrane approach as a way to obtain sweet and unsweet lactose-free milk
Source: Bioprocess Biosyst Eng. 2024 Apr 22;47(6):919–29. doi: 10.1007/s00449-024-03018-z (PMC11101535; doi:10.1007/s00449-024-03018-z)
Supplement: Supplementary file 1 — Supplementary file1 (DOCX 122 KB) [file 449_2024_3018_MOESM1_ESM.docx]

**Supplementary Information for**

**A catalytic membrane approach as a way to obtain sweet and unsweet lactose-free milk**

Katarzyna Czyżewska^1*^, Anna Trusek^1^

^*^corresponding author

^1^Wroclaw University of Science and Technology, Faculty of Chemistry, Group of Micro, Nano, and Bioprocess Engineering

ul. Norwida 4/6, 50-373 Wrocław, Poland

Tel. +48 71 320 33 14

email: katarzyna.czyzewska@pwr.edu.pl


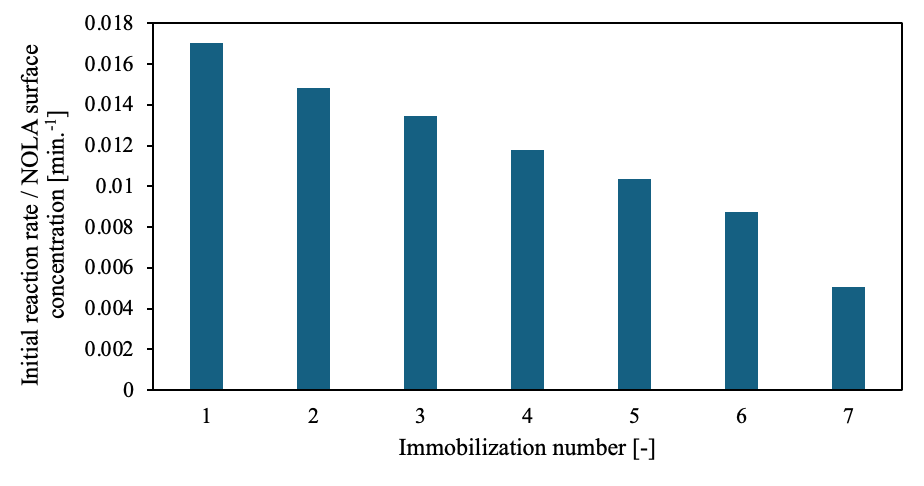


Figure S1. The quality of catalytic membranes made on RC with covalently bonded NOLA, created by reusing the Na_2_CO_3_/NaHCO_3_ solution with the enzyme. Lactose concentration of 55 g/L, average surface concentration of NOLA 5.54 g/m^2^, 0.1M HEPES buffer pH 6.6, 15°C.

.

Table S1. Lactose hydrolysis catalyzed by immobilized NOLA on PA membrane. Lactose 55 g/L, 0.1M HEPES buffer pH 6.6, 15°C, GA 2.5%, supplied mass of NOLA 73.26 mg, surface concentration 3.65 g/m^2^, immobilization yield 2.44%.

| time [h] | glucose concentration [g/L] | conversion yield [%] |
| --- | --- | --- |
| 0 | 0 | 0. |
| 0.17 | 0.01 | 0.05 |
| 0.5 | 0.02 | 0.09 |
| 1 | 0.03 | 0.14 |
| 2 | 0.04 | 0.16 |
| 2.5 | 0.06 | 0.24 |
| 5 | 0.07 | 0.30 |
| 24 | 0.11 | 0.42 |
| 29 | 0.11 | 0.45 |
| 48 | 0.14 | 0.57 |

Table S2. Lactose hydrolysis catalyzed by immobilized NOLA on PA membrane. Lactose 55 g/L, 0.1M HEPES buffer pH 6.6, 15°C, GA 1%, supplied mass of NOLA 68.5 mg, surface concentration 1.63 g/m^2^, immobilization yield 1.17%.

| time [h] | glucose concentration[g/L] | conversion yield [%] |
| --- | --- | --- |
| 0 | 0 | 0 |
| 0.17 | 0.00 | 0.01 |
| 0.5 | 0.01 | 0.05 |
| 1 | 0.02 | 0.07 |
| 2 | 0.04 | 0.15 |
| 2.5 | 0.06 | 0.22 |
| 5 | 0.06 | 0.25 |
| 24 | 0.11 | 0.45 |
| 29 | 0.12 | 0.48 |
| 48 | 0.13 | 0.51 |


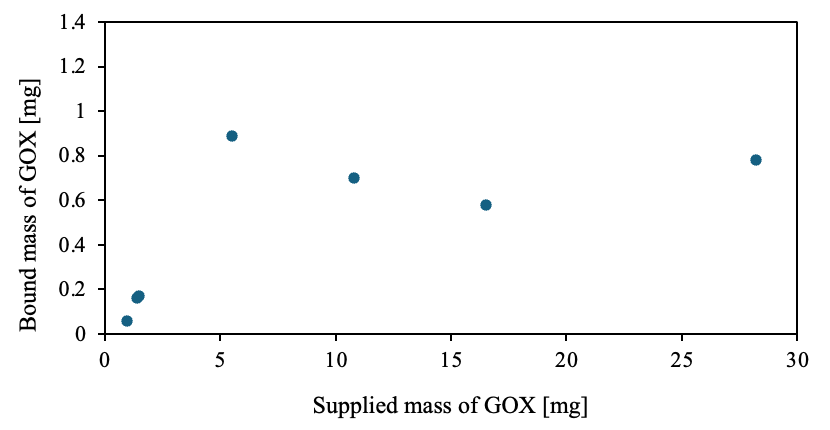


Figure S2. The efficiency of forming catalytic membranes with immobilized GOX on RC. Membrane area of
4.9 cm^2^, enzyme surface concentration in the range of 0.12 to 1.82 g/m^2^.


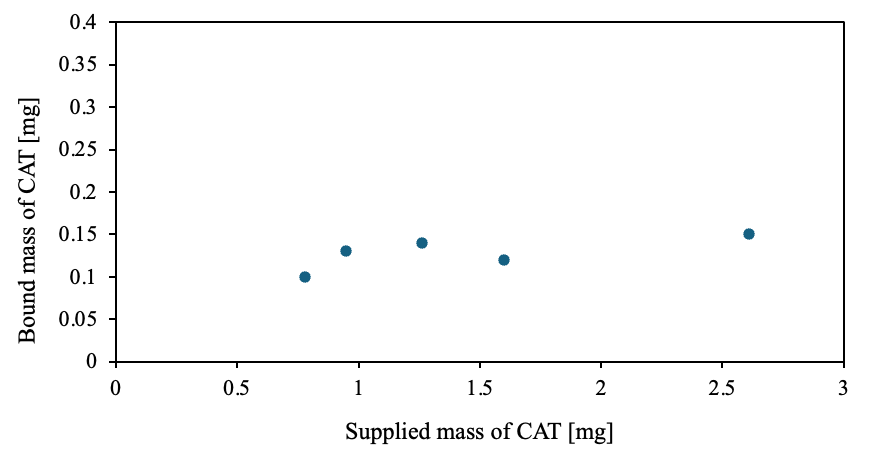


Figure S3. The efficiency of forming catalytic membranes with immobilized CAT on RC. Membrane area of
4.9 cm^2^, enzyme surface concentration in the range of 0.2 to 0.347 g/m^2^.
